# Study on the Effects of Blending Basalt Fiber and Polyethylene Fiber on the Mechanical Properties and Microstructure of Mortars

**DOI:** 10.3390/ma19050881

**Published:** 2026-02-27

**Authors:** Jian Gong, Wenwen Zhao, Qian Liu, Qingfeng Chen, Huazhe Jiao, Liuhua Yang, Weizhun Jin

**Affiliations:** 1 School of Civil Engineering, Zhengzhou University of Technology, Zhengzhou 450044, China; gongjian890@163.com (J.G.); zhaowenwen2026@163.com (W.Z.); 2 School of Civil Engineering and Transportation, North China University of Water Resources and Electric Power, Zhengzhou 450045, China; liuqian06020@163.com; 3 College of Civil Engineering, Henan University of Engineering, Zhengzhou 451191, China; 4 School of Civil Engineering, Henan Polytechnic University, Jiaozuo 454003, China; jiaohuazhe@126.com (H.J.); yanglh2005@163.com (L.Y.)

**Keywords:** basalt fiber, polyethylene fiber, blended fibers, mechanical strength, microstructure

## Abstract

Fiber reinforcement technology has become one of the effective ways to improve the mechanical properties and deformation capacity of concrete. This study investigated the effects of single-doped and blended-doped basalt fiber (BF) and polyethylene fiber (PEF) on the drying shrinkage and mechanical strength of mortars. Meanwhile, the microstructure and reinforcement mechanism of single-doped and blended-doped BF and PEF mortars were studied. The results show that the mortar with a single-doped 6 mm PEF has the strongest resistance to drying shrinkage, and that blended fibers also effectively enhance the resistance to drying shrinkage of mortars. The compressive strength and flexural strength of the blended-fiber mortars are both higher than those of the single-fiber mortar. When the fiber length was 12 mm and the BF/PEF was 1:1, the compressive strength and flexural strength of the mortar at 28 d were respectively 18.6% and 56.1% higher than those of the mortar without fiber. Furthermore, when the fiber lengths were both 12 mm and 18 mm, the splitting tensile strength of the blended-fiber mortar at 28 d was higher than that of the single-fiber mortar and the mortar without fiber. When the fiber length was 12 mm and the BF/PEF was 1:1, the splitting tensile strength of the blended-fiber mortar was 103.3% higher than that of the mortar without fiber. The BF is randomly distributed in the mortar in the form of single filaments, and it exhibits brittle fracture when the mortar fails. When the mortar is damaged, PEF exhibits the phenomenon that the fibers are pulled out, and its surface is covered with hydration products, demonstrating excellent interfacial bonding performance. BF and PEF can interlock and intertwin with each other, forming a three-dimensional network structure in mortar, and jointly exert a complementary reinforcing effect of rigidity and flexibility.

## 1. Introduction

Ordinary concrete holds a significant position in structural engineering due to its low cost, high compressive strength, and good durability [1]. However, ordinary concrete is a brittle material, and its ability to stretch is relatively poor. When subjected to tension or bending, it is prone to sudden fracture, and the cracks expand rapidly and are difficult to control. These inherent flaws severely restrict the application of concrete in certain projects with high toughness or strict crack resistance requirements [2].

To improve the crack resistance and deformation performance of cement-based materials, fiber reinforcement technology has been widely adopted [3]. After the fibers are uniformly dispersed in the cement matrix, they can span the crack development area, exert a bridging effect, and prevent stress concentration, thereby effectively delaying the initiation and expansion of cracks. This mechanism of action can significantly improve the fracture properties of cement-based materials, enabling them to exhibit greater energy absorption capacity during failure, and it can also help alleviate the internal stress concentration caused by drying shrinkage and temperature changes [4,5]. At present, the commonly used fibers in cement-based materials mainly include steel fiber (SF) [6,7], glass fiber (GF) [8,9], basalt fiber (BF) [10,11], polyethylene fiber (PEF) [12,13], polyvinyl alcohol fiber (PVAF) [14,15], and carbon fiber (CF) [16,17], etc. The incorporation of these fibers can effectively improve the mechanical properties of cement-based materials and have significant effects in controlling crack width and enhancing deformation capacity and impact resistance [18].

However, fibers of a single type or size are often capable of providing only limited enhancement effects in terms of specific scales or mechanical properties [19]. Studies have shown that the use of mixed fibers can achieve synergy and complementarity among different fibers at different stages of crack development, thereby enabling the control of the entire cracking process. Generally, the fine and short fibers can play a role in preventing cracks at the micro-crack stage, while the thicker and longer fibers can provide strong bridging after the cracks expand, thereby jointly ensuring the effective transmission of stress between the cracks [20,21]. Wang et al. [22] investigated the influence of fiber distribution on the mechanical properties of steel-PVA hybrid fiber-reinforced engineering cement-based composites (ECCs). The results show that the mixed fibers exhibit a significant synergistic effect in controlling the width of tensile cracks. The incorporation of steel fibers significantly enhanced the crack control capability. Moreover, when the volume fraction of steel fibers reached 1.0%, the average crack width decreased to 36.1 μm. Zhang et al. [23] systematically evaluated the influence of the proportion of polypropylene fiber (PPF)-BF hybrid fibers on the mechanical properties of concrete. The results show that when PPF are mixed with BF at a ratio of 1.0% + 1.0%, the concrete exhibits the best overall performance and demonstrates a higher flexural-to-compressive ratio. The blended fiber concrete can achieve a tensile strength of 3.7 MPa and a tensile strain of 2.0%.

BF is made by drawing basalt melt and features high strength, high elastic modulus, and good chemical resistance and stability. BF can significantly enhance the tensile strength, flexural strength, and deformation resistance of concrete, and can effectively inhibit concrete cracking [24,25]. Xu et al. [26] conducted experiments to explore the feasibility of preparing ECCs using BF. The results show that BF-ECC exhibits unique strain hardening and multi-groove cracking characteristics. Its tensile stress–strain curve is relatively smooth, and the average crack width is less than 10 μm. Wang et al. [27] found that the incorporation of BF could increase the 3-day flexural strength of the mortar by 27%, and the toughness index was 6.5 times that of the control group. PEFs have high tensile strength and a high elongation rate, thus possessing good toughness and ductility, which can effectively enhance the crack resistance of concrete [28,29,30]. Kim et al. [29] investigated the effect of hybrid SF and PEF on the mechanical performance of UHPC and found that the strain capacity and energy absorption capacity per unit volume significantly increased with the addition of PEF. Xie et al. [30] studied the effect of hybrid SF and PEF on the compressive behavior of cementitious composite and revealed that the compressive strength, peak strain, and residual stress of the cementitious composite were significantly improved by hybrid SF and PEF. Therefore, blending BF with PEF is expected to enable the concrete to possess both high strength and excellent toughness.

The current research by scholars mainly focuses on the studies of single fibers or different proportions of mixed fibers. However, the impact of different fiber lengths and different proportions of mixed fibers on the drying shrinkage performance, mechanical properties, and microstructure of concrete have not been fully explored. Based on this, the purpose of this study is to investigate the synergistic effects of different lengths (6 mm, 12 mm, and 18 mm) of BF and PEF, as well as the influence of different volume ratios (BF:PEF = 3:1, 1:1, and 1:3) on the mechanical properties and microstructure of mortar. The drying shrinkage, compressive strength, flexural strength, and splitting tensile strength of blended-fiber concrete were tested. Additionally, the strain evolution and crack propagation laws of the blended-fiber mortar during the splitting tensile process were analyzed using digital image correlation (DIC) technology. Furthermore, SEM was used to observe the microscopic morphology of blended-fiber mortar, revealing the synergistic mechanism of BF and PEF in the mortar. This research aims to provide a theoretical basis and experimental support for the design of high strength and high crack resistance concrete.

## 2. Materials and Methods

### 2.1. Materials

**Figure 1:**
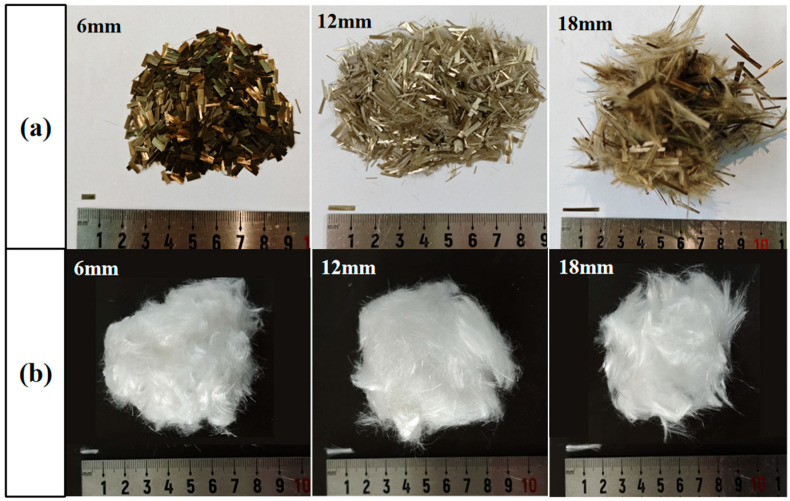
Appearance morphologies of fibers: (**a**) BF; (**b**) PEF.

In this study, ordinary Portland cement (OPC) with a strength grade of 42.5 was used as the cementitious material, and its chemical composition is shown in Table 1. River sand with a fineness modulus of 2.76 was selected as the fine aggregate. The water reducer used was the polymeric carboxylic acid high-performance powder water reducer produced by Shanxi Feike New Materials Technology Co., Ltd. (Yuncheng, China). Tap water was used as the mixing water. BF and PEF were provided by Huimin County Taili Textile Products Co., Ltd. (Binzhou, China). The performance parameters of BF and PEF are shown in Table 2, and their appearance morphologies are illustrated in Figure 1.

### 2.2. Mix Design

The mix ratio of the fiber reinforced mortar is shown in Table 3. The cement-to-sand ratio of the fiber-reinforced mortar is 1:3, and the water-cement ratio is 0.5. According to previous studies [31,32], when the volume content of fibers in cement-based materials is around 1.0% by volume, the cement-based materials will exhibit better mechanical properties. Therefore, in this study, the fiber volume fraction of the single-doped fiber group is 1.0% by volume. The total volume fraction of the BF and PEF blended group is fixed at 1.0% by volume. The ratios of the two fibers are BF:PEF = 3:1, 1:1, and 1:3.

### 2.3. Sample Preparation and Curing

**Figure 2:**
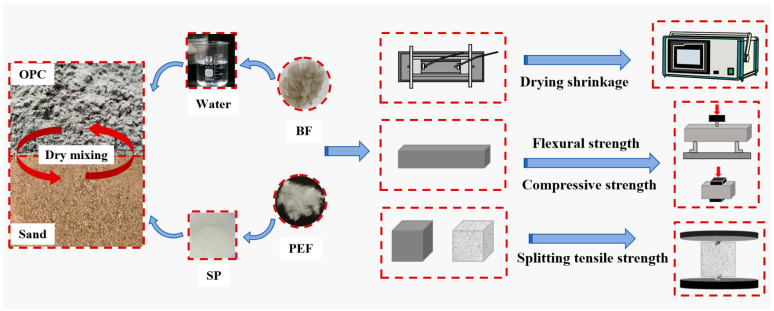
Preparation process of fiber-reinforced mortars, and tests of physical and mechanical properties.

The preparation process of the fiber-reinforced mortar is shown in Figure 2. First, add the cement and sand to the mixer and stir at 140 r/min for 3 min to ensure that the dry materials are thoroughly mixed. Then, add the water containing PS and stir at 140 r/min for 3 min. Next, slowly add BF and PEF to the slurry, and stir the mixture at 140 r/min for 3 min to obtain a mortar with uniformly dispersed fibers. Then, pour the freshly mixed mortar into the mold and compact it firmly. Finally, naturally cure the mortar for 24 h and then remove it from the mold. Then, place it in a standard curing room (20 ± 2 °C, RH ≥ 95%) for 28 d.

### 2.4. Test Methods

According to Chinese standard GB/T 29417-2012 [33], the drying shrinkage test of fiber-reinforced mortar was conducted. The lengths of the fiber-reinforced mortar were measured at 3 d, 7 d, 14 d, 21 d, and 28 d, and the drying shrinkage at each stage was calculated. The average value of three samples from each group was taken as the final drying shrinkage value of the fiber-reinforced mortar.

According to Chinese standard GB/T 17671-2021 [34], the compressive and flexural strengths of fiber-reinforced mortar were tested. The size of the mortar blocks used for the compressive and flexural strength tests was 40 mm × 40 mm × 160 mm. The loading rate during the test process was 50 N/s. The average value of the three samples in each group was taken as the flexural strength. Then, the six mortar blocks after the flexural strength test were subjected to a compressive strength test. The loading rate during the test process was 2.4 kN/s. The average value of the six samples in each group was taken as the compressive strength. The splitting tensile strength of the fiber-reinforced mortar was tested in accordance with Chinese standard GB/T 50081-2019 [35]. In the splitting tensile strength test, the size of mortar block was 70.7 mm × 70.7 mm × 70.7 mm and the loading rate was 0.05 MPa/s.

DIC technology was employed to study the variation pattern of cracks in the mortar during the splitting tensile process. Before the test, speckles were created on the surface of the mortar block. During the testing process, images were recorded through the camera system. Finally, the collected images were analyzed using Ncorr1.0 developed based on Matlab software to obtain the strain field of the mortar.

The microscopic structure of the fiber–matrix interface was observed via SEM. Before the test, the mortar fragments obtained from the compressive strength test were placed in a 60 °C oven for drying. Then, the surface of the sample was coated with gold. Finally, the samples were subjected to vacuum treatment and their microstructures were observed.

## 3. Results and Discussion

### 3.1. Drying Shrinkage

**Figure 3:**
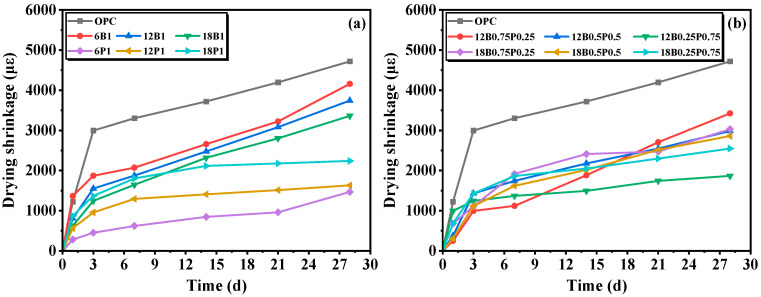
Dry shrinkage properties of mortars incorporating fibers: (**a**) Mortars incorporating different single fiber; (**b**) Mortars incorporating blended fibers.

The drying shrinkage of mortars mixed with different types and lengths of fibers is shown in Figure 3. As shown in Figure 3a, compared with the control group without fiber, the addition of BF and PEF with a volume fraction of 1.0 vol% can effectively reduce the drying shrinkage of mortars. Compared with the control group without fiber, the mortars containing 6 mm, 12 mm, and 18 mm of BF show a reduction in shrinkage values of 11.9%, 20.7%, and 28.8%, respectively, at 28 d. It can be seen that the addition of BF can effectively enhance the shrinkage resistance of mortar. This is mainly attributed to the bridging effect formed by BF within the matrix, which can enhance the bonding stress between fibers and the matrix, thereby effectively inhibiting the formation and development of microcracks and reducing the drying shrinkage of the mortar [36]. Furthermore, when the BF content is 1.0% by volume, the longer BF has a stronger resistance to drying shrinkage. The drying shrinkage value of the mortar mixed with 18 mm BF is 19.2% lower than that of the mortar mixed with 6 mm BF. This is because longer BFs are more likely to interlock with each other and form a continuous and robust three-dimensional network structure within the matrix, providing comprehensive constraints for the matrix and significantly enhancing the overall integrity of the mortar to resist deformation. Conversely, shorter BF has a limited bridging range and its bonding with the matrix is relatively weak, making it difficult to form an efficient spatial network structure. This will result in the restraint effect of the fibers being limited to a local area and being relatively scattered, thereby weakening the overall inhibitory effect of the fibers on the drying shrinkage of mortars [37]. Similarly, the mortars containing 6 mm, 12 mm, and 18 mm of PEF show a reduction in the dry shrinkage value of 68.9%, 65.4%, and 52.6%, respectively, compared to the control group at 28 d. When the content of PEF is 1.0% by volume, the shorter PEF exhibits better anti-shrinkage performance. The drying shrinkage value of the mortar containing 6 mm PEF is 34.4% lower than that of the mortar containing 18 mm PEF. This is mainly due to the fact that the longer flexible PEF is prone to bending and agglomeration during the mixing and hardening of the mortar, making it difficult to exert its restraining effect. Meanwhile, the bent PEF in the mortar needs to be straightened under force, and this process consumes relatively little energy, thereby reducing the efficiency of the fibers in constraining the matrix [38]. Furthermore, the aggregation of PEF may introduce defects and water channels into the matrix, leading to the further development of local microcracks, thereby weakening the inhibitory effect of fibers on the drying shrinkage of mortar to a certain extent [39].

As can be seen in Figure 3b, when the total volume fraction of BF and PEF is 1.0% by volume, the ratio of the fiber length to the mixed fiber can significantly affect the shrinkage resistance performance of the mortar. When the fiber lengths are all 12 mm, the shrinkage values of the mortars mixed with the blended fibers BF/PEF at ratios of 3:1, 1:1, and 1:3 at 28 d are respectively 27.5%, 36.8%, and 60.5% lower than those of the control group. Meanwhile, the 12B0.25P0.75 group exhibits the most effective inhibition on the drying shrinkage of the mortar. This indicates that when the fiber length is 12 mm, increasing the proportion of flexible PEF fibers helps to enhance the overall restraint of the mortar [40]. When the fiber lengths are all 18 mm and the blending fibers BF/PEF are mixed in at ratios of 3:1, 1:1, and 1:3, the shrinkage values of the mortar after 28 d are 35.8%, 39.4%, and 46.0% lower than those of the control group, respectively. Meanwhile, the 18B0.25P0.75 group exhibits the best inhibitory effect on the drying shrinkage of mortar. This further confirms that a higher content of PEF has a stronger anti-drying shrinkage advantage when longer fibers are added to the mortar.

### 3.2. Compressive Strength

**Figure 4:**
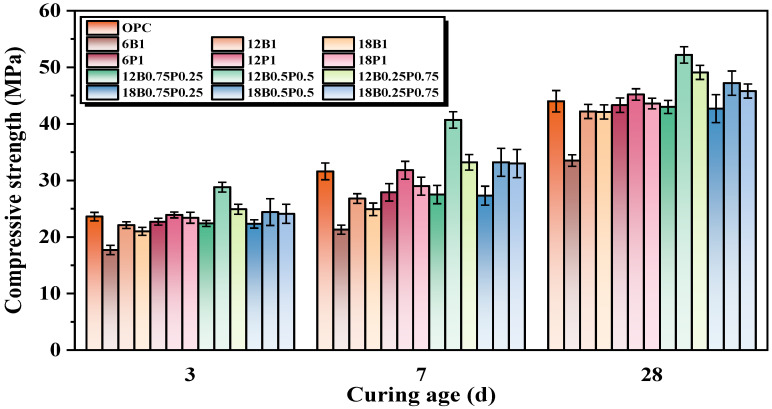
Compressive strength of mortars with single and blended incorporation of various lengths of BFs and PEFs.

The compressive strength of mortars with single and mixed additions of different types and lengths of BF and PEF are shown in Figure 4. It can be seen that when the BF dosage is 1.0% by volume, the compressive strength of all the mortars with BF is lower than that of the mortars without fibers. Compared with the mortar without fiber, the compressive strength of the mortar mixed with 6 mm, 12 mm, and 18 mm BF alone decreases by 23.9%, 4.1%, and 4.3%, respectively, at 28 d. This is mainly attributed to the poor dispersion of the higher fiber content in the matrix, which is prone to agglomeration and forms local weak areas within the mortar, thereby adversely affecting its compressive strength [27]. Furthermore, the compressive strength of the mortar mixed with 6 mm BF decreases most significantly. This is mainly due to the fact that the aspect ratio of shorter fibers is relatively small, making it difficult for them to effectively transfer loads within the mortar matrix. Moreover, this may increase the number of initial defects, thereby leading to a significant reduction in strength. In contrast, the 12 mm and 18 mm BFs can effectively bridge the micro-cracks within the mortar and inhibit the development of cracks, thereby compensating to some extent for the adverse effects caused by the introduction of fibers [41]. Furthermore, PEF demonstrates a superior reinforcing effect on the mortar. Compared with the mortar without fiber addition, the mortars with 6 mm and 18 mm PEFs alone have a 1.6% and 0.9% reduction in compressive strength at 28 d, respectively, while the mortar with 12 mm PEF alone has a 2.7% increase in compressive strength at 28 d. It can be seen that the compressive strength of the mortars mixed with 6 mm and 18 mm PEF has slightly decreased. This is mainly related to the spatial distribution of the fibers within the mortar matrix and the efficiency of load transmission. The shorter PEF (6 mm) has a limited ability to bridge cracks, while the longer PEF (18 mm) is prone to bending and agglomeration during the mortar mixing process, resulting in uneven dispersion and thereby weakening its reinforcing effect on the mortar. Furthermore, the longer PEF tends to cause an expansion of the interface transition zone (ITZ) between fibers and the matrix and it becomes a stress concentration area, resulting in a further decrease in the compressive strength of the mortar [42].

When the total volume fraction of the mixed fibers is 1.0% by volume, the improvement in the compressive strength of mortar by blended fibers is better than that of the single fiber. When the lengths of BF and PEF are both 12 mm, and the ratio of BF to PEF is 3:1, the compressive strength of mortar after 28 d is 2.3% lower than that of the mortar without a fiber addition. This is because when the proportion of BF is high, it is prone to agglomeration within the matrix, forming local weak areas. Moreover, the bonding performance at the fiber–matrix interface is insufficient, thereby reducing the bearing capacity of the mortar. When the lengths of the BF and PEF are both 12 mm, and the ratios of BF to PEF are 1:1 and 1:3, the compressive strength of mortar at 28 d increases by 18.6% and 11.6%, respectively, compared to the mortar without a fiber addition. Meanwhile, when the BF/PEF ratio is 1:1, the compressive strength of mortar at 28 d is 23.7% and 15.5% higher than that of the mortar with only 12 mm BF and PEF added, respectively. This is because the increase in the proportion of PEF can effectively improve the dispersion of BF. Meanwhile, PEF has high elongation at break and excellent toughness. It can absorb a large amount of energy after the matrix cracks and bridge wide cracks through plastic deformation to prevent the cracks from penetrating [43]. When the lengths of BF and PEF are both 18 mm and the ratios of BF/PEF are 3:1, 1:1, and 1:3 respectively, the growth trend of the compressive strength of the mortar at 28 d is consistent with that when the fiber length is 12 mm, and both reach the highest value when the ratio of BF/PEF is 1:1. Therefore, the blending of BF with PEF can effectively optimize the stress distribution of the mortar when subjected to loads, inhibit the development of cracks, and significantly enhance the compressive strength of the mortar.

### 3.3. Flexural Strength

**Figure 5:**
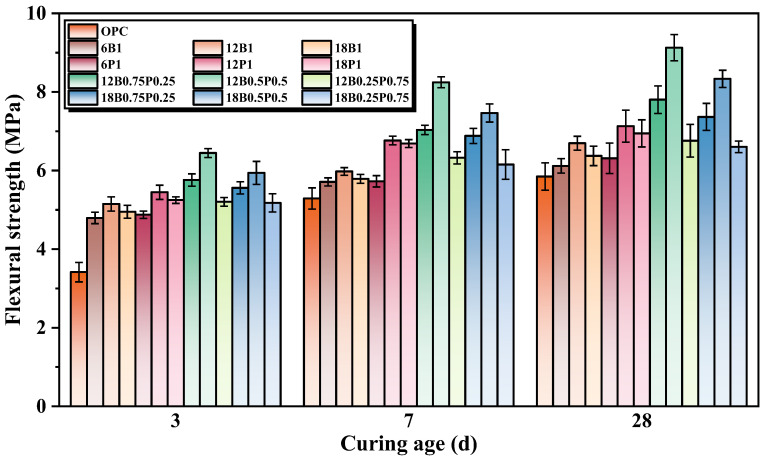
Flexural strength of mortars with individual and hybrid incorporation of various lengths of BFs and PEFs.

The flexural strength of mortars mixed with different types and lengths of BF and PEF in single-doped and blended-doped forms is shown in Figure 5. Compared with the mortar without added fibers, the addition of 1.0% by volume of a single type of fiber has an enhancing effect on the flexural strength of mortar. Compared with the mortar without fibers, the mortars with 6 mm, 12 mm, and 18 mm of BF added alone increase their flexural strength by 4.6%, 14.5%, and 8.9%, respectively, at 28 d. It can be seen that the 12 mm BF has the most significant enhancing effect on the flexural strength of mortar, while the 6 mm and 18 mm BFs have a relatively lower enhancing effect on the flexural strength of mortar. This is because BF can form chemical bonds with the hydrated products, and BF also has a relatively high elastic modulus. When the length of BF is increased from 6 mm to 12 mm, its bridging capability can be fully utilized. When the BF length is 18 mm, it is prone to agglomeration in the mortar, forming local defects, which leads to a decrease in its pull-out strength. Furthermore, compared with the mortar without fibers, the mortars with 6 mm, 12 mm, and 18 mm of PEF added alone increase their flexural strength by 7.9%, 21.9%, and 18.8%, respectively, at 28 d. This is mainly attributed to the high toughness and excellent load transfer capability of PEF, which enables it to effectively delay the development of cracks during tension and significantly enhance the ductility and toughness of the matrix. The flexural strength of the mortar containing 12 mm PEF at 28 d increases by 13.0% and 2.6%, respectively, compared to the mortars containing 6 mm and 18 mm PEFs. This is because the influence of fiber length on tensile strength mainly stems from the balance between the bridging effect and the dispersion of the fibers. Although the ultra-short fibers (6 mm) can be uniformly dispersed in the matrix, their effective anchoring length is insufficient, making them prone to being pulled out from the matrix under force. In contrast, the longer fibers (18 mm) tend to entangle and aggregate within the mortar, forming defects, which in turn weakens its stress transmission capacity [44]. The moderate length (12 mm) can be evenly dispersed in the mortar and can fully bond with the substrate, increasing the friction between the fibers and the substrate, thereby effectively crossing the cracks and achieving the optimal bridging effect [45].

When 1.0% by volume, different lengths and proportions of BFs and PEFs exhibit a significant synergistic strengthening effect on the flexural strength of mortar. When the lengths of the BF and PEF are both 12 mm, and the ratios of BF to PEF are 3:1, 1:1, and 1:3, the flexural strengths of the mortar at 28 d increase by 33.3%, 56.1%, and 15.6%, respectively, compared to the mortar without fiber addition. This is mainly due to the fact that during the synergistic reinforcement process of BF and PEF, BF has a higher elastic modulus and tensile strength, which can effectively bridge the macroscopic cracks and prevent the development of cracks. PEF possesses high ductility and fracture energy, which can effectively inhibit the initiation and propagation of microcracks, as well as disperse the stress at the tip of the cracks. After blending BF and PEF, a dense three-dimensional network structure can be formed in the matrix, which delays the crack propagation and enhances the toughness and load-bearing capacity of the matrix, presenting a significant positive hybrid effect [46]. When the ratios of BF/PEF are 3:1 and 1:3, the flexural strength of the mortar decreases compared to when the ratio of BF/PEF is 1:1. This is mainly attributed to the weakened synergy resulting from the imbalance in the proportion of fibers. When the BF content is too high, it leads to a decrease in the dispersion of the fibers, making it prone to forming local weak areas in the mortar. When the proportion of PEF is too high, the overall stiffness of the mortar is insufficient, which inhibits its ability to restrain macroscopic cracks. When the lengths of BF and PEF are both 18 mm, and the ratios of BF/PEF are 3:1, 1:1, and 1:3, the flexural strength of the mortars at 28 d increase by 26.0%, 42.4%, and 12.8%, respectively, compared to the mortar without a fiber addition. It can be seen that although the reinforcing effect of the blended fiber system with a fiber length of 18 mm is generally weaker than that of the blended fiber system with a fiber length of 12 mm, it is still generally superior to that of the single fiber system.

### 3.4. Splitting Tensile Strength

**Figure 6:**
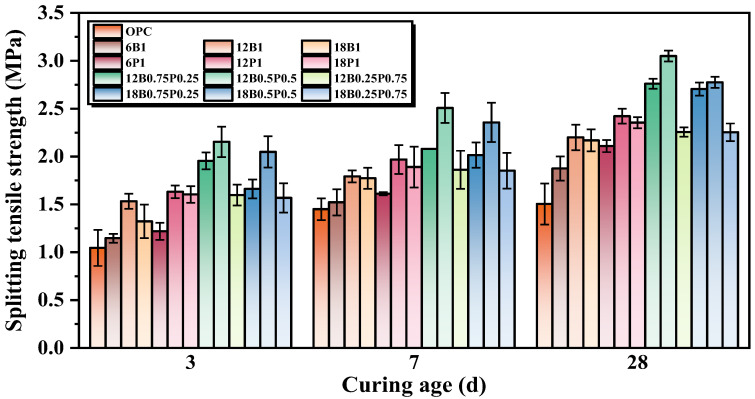
Splitting tensile strength of mortars with individual and hybrid incorporation of various lengths of BF and PEF.

The splitting tensile strengths of mortars composed of different types and lengths of BFs and PEFs are shown in Figure 6. It can be seen that for single-fiber reinforced concrete, regardless of whether BF or PEF is added, the splitting tensile strength of the mortar increases first and then decreases as the fiber length increases. The splitting tensile strengths of the mortars with a single addition of 6 mm, 12 mm, and 18 mm BF at 28 d increase by 24.7%, 46.7%, and 44.7%, respectively, compared to the mortar without a fiber addition. It can be seen that the 12 mm BF has the greatest effect in enhancing the splitting tensile strength of the mortar. This is because the 12 mm BF can more effectively bridge cracks in the mortar and transfer stress, thereby significantly enhancing the splitting tensile strength of the mortar [47]. Meanwhile, the longer fiber (12 mm) exhibits a stronger bridging and uplift resistance in the mortar compared to the shorter fibers (6 mm), and contributes more to the enhancement of the mortar’s splitting tensile strength [48]. However, overly long fibers (18 mm) have a negative impact on the compactness of the mortar due to their entanglement, bending, and uneven dispersion. At the same time, overly long fibers can lead to local stress concentration, which is not conducive to enhancing the strength of mortars [49]. The mortars containing 1.0% of 6 mm, 12 mm, and 18 mm PEF have a 40.7%, 61.3%, and 56.7% higher splitting tensile strength at 28 d compared to the mortar without fiber addition, respectively. It can be seen that the splitting tensile strength of the mortars containing 12 mm and 18 mm fibers is nearly the same, and both are significantly higher than that of the mortar containing 6 mm fibers. This is because the shorter PEF (6 mm) is prone to be pulled out under load, and its reinforcing effect is limited. The longer PEFs (12 mm and 18 mm) can effectively disperse stress by means of bridging action and their own large deformation, thereby inhibiting the development of cracks due to their high flexibility and ductility [50]. Furthermore, longer fibers (18 mm) are more prone to buckling when subjected to force, which to some extent reduces the efficiency of stress transmission, resulting in a slightly less significant enhancement in the mortar strength compared to the 12 mm fibers.

When the lengths of the BF and PEF are both 12 mm, and the ratios of BF/PEF are 3:1, 1:1, and 1:3, respectively, and the splitting tensile strengths of mortars at 28 d increase by 84.0%, 103.3%, and 50.7%, respectively, compared to the mortar without a fiber addition. It can be seen that when the ratio of BF/PEF is 1:1, the addition of blending fibers achieves the best improvement in the splitting tensile strength of mortar, which is 26.0% higher than that of the mortar with only PEF added. This is because the elastic modulus of BF is relatively high, which can effectively prevent the formation and expansion of microcracks, thereby enhancing the early stiffness of the mortar. Meanwhile, PEF has excellent toughness and a high elongation at break, which enables it to continuously provide bridging effects and dissipate energy during the later stage of crack propagation. Therefore, after blending BF and PEF, a continuous and efficient stress transmission system is formed at different stages of crack development, thereby achieving an enhanced effect that surpasses that of a single fiber. Furthermore, when the fiber length is 18 mm and the ratios of BF/PEF are 3:1, 1:1, and 1:3, the splitting tensile strengths of mortars at 28 d increases by 50.7%, 84.7%, and 50.0%, respectively, compared to the mortar without fiber addition. It can be seen that although the splitting tensile strength of the mixed fiber mortar is slightly lower than that of the mortar with 12 mm fibers alone, it is significantly higher than that of the mortar without fibers and than most of the mortars with only 6 mm and 18 mm fibers added. This is because although the longer fibers (18 mm) have a wider bridging range in the mortar, they are also more prone to bending and uneven distribution, resulting in a slightly lower enhancement efficiency compared to the 12 mm fibers. However, the functional complementarity of stiffness and toughness between the BF and PEF still enables the effective enhancement of the splitting tensile strength of the mortar.

### 3.5. Analysis of the Failure Process of Mortar

**Figure 7:**
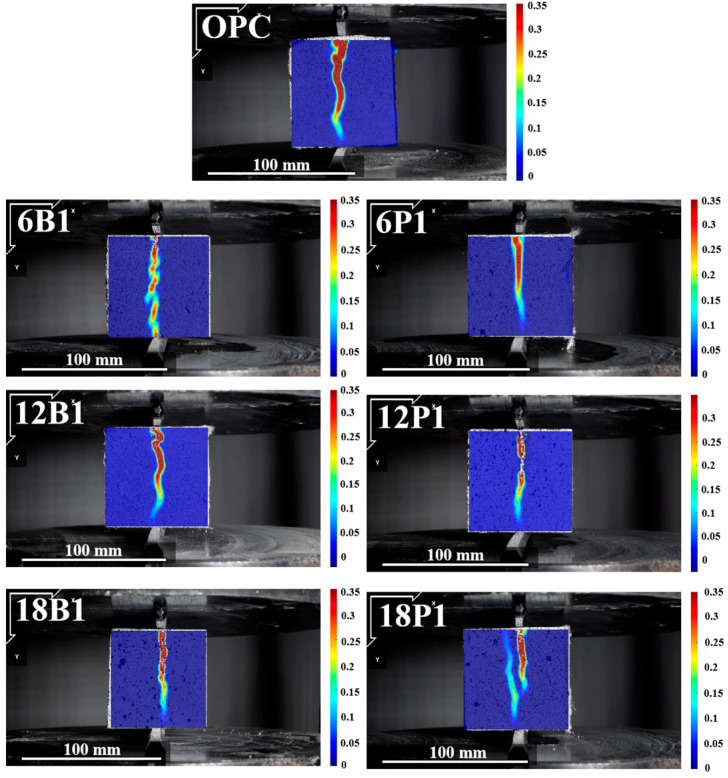
Strain distribution of mortars with single fiber type and varying lengths under splitting tension.

The crack changes in mortars with a single addition of BFs and PEFs of different lengths during the splitting failure process are shown in Figure 7. For the mortar with a single addition of 6 mm BF, during the failure process, it exhibits obvious local crack propagation and the crack width is relatively wide, concentrating in the middle part of the sample. As the length of the BF increases to 12 mm, the crack width of the mortar decreases but the overall stress concentration trend of the mortar still does not show significant improvement. When the length of the BF increases to 18 mm, the crack width of the mortar further decreases. However, due to the influence of the BF stiffness, the local stress concentration phenomenon of the mortar still exists. This indicates that the BF with a higher elastic modulus can play a role in bridging the cracks and effectively inhibit crack propagation, but that its ability to disperse stress concentration is limited [51]. In contrast, the crack propagation depth of the mortar containing PEF is significantly shallower than that of the mortar containing BF, demonstrating a more superior crack suppression ability. Furthermore, when longer PEFs (12 mm and 18 mm) are added to the mortar, the width of the cracks in the mortar becomes significantly narrower. Meanwhile, when the PEF is 18 mm, two fine cracks appear in the mortar. This is attributed to the high flexibility and ductility of PEF, which enables it to stretch and undergo interface slip during the force application process. This allows the stress to be redistributed over a larger area, effectively delaying the local development of cracks and enhancing the tensile strength and toughness of the mortar [52].

**Figure 8:**
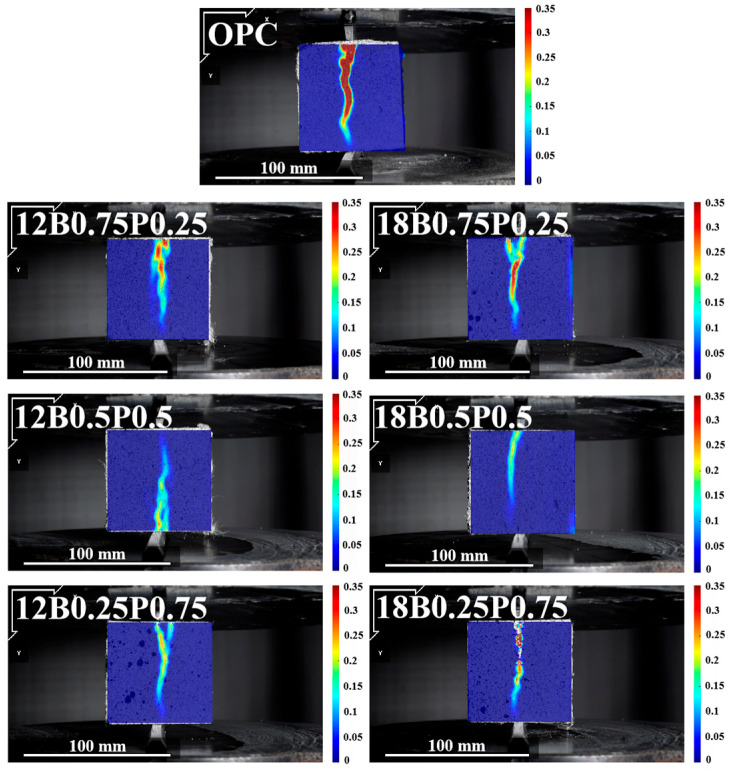
Crack propagation of mortars with blended fibers at various lengths and proportions under splitting tension.

The changes in cracks during the splitting failure process of mortars mixed with different lengths and proportions of BFs and PEFs are shown in Figure 8. According to the DIC cloud diagram, it can be observed that the ratio of BF to PEF has a significant impact on the crack propagation pattern of mortar. As the proportion of PEF in the mixture gradually increases, the depth of the cracks in the mortar gradually becomes shallower and narrower. This is mainly attributed to the high flexibility and tensile deformation capacity of PEF, which enables it to dissipate stress over a larger area during tension by means of its own slip and ductile deformation, thereby effectively inhibiting the development of local cracks [53]. Furthermore, increasing the fiber length from 12 mm to 18 mm can significantly enhance the properties of all the blended fiber mortars. Moreover, when the proportion of PEF is higher, the reinforcing effect of the blended fibers becomes more pronounced. The crack propagation width of the 18 mm fiber-reinforced mortar is less than that of the 12 mm fiber-reinforced mortar, indicating that the longer PEF has a stronger bridging ability and can significantly inhibit the crack propagation of the mortar.

### 3.6. SEM

**Figure 9:**
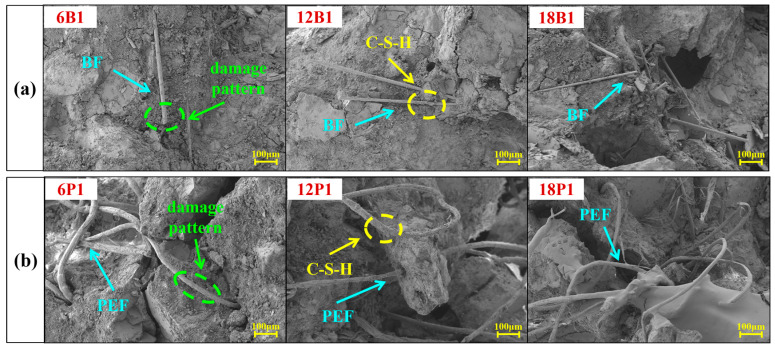
SEM images of mortars incorporating different single fiber: (**a**) Mortars incorporating different length of BF; (**b**) Mortars incorporating different length of PEF.

The microscopic morphologies of mortars with single addition of different types and lengths of BF and PEF are shown in Figure 9. Figure 9a and b respectively represent the microscopic morphologies of the mortars doped with 1.0% BF by volume and 1.0% PEF by volume. It can be seen that the surfaces of both the BF and PEF are covered by hydration products, indicating that a good interface bonding has been formed between the fibers and the matrix, which has a significant impact on the strengthening, toughening, and crack resistance of the mortar [54]. In Figure 9a, BF is randomly distributed in the mortar in the form of single filaments, which can effectively inhibit the expansion of microcracks [55]. Meanwhile, no obvious wear marks are observed on the BF surface, and the end part exhibits the typical brittle fracture morphology. This is because the hydrophilic nature of BF endows it with excellent bonding ability, which helps to enhance the cracking resistance of the mortar when subjected to loads, and delays the occurrence and development of cracks [56,57]. Furthermore, the shorter 6 mm BF is prone to be pulled out under force, making it difficult to fully utilize its high elastic modulus and high tensile strength. The 12 mm and 18 mm BFs are capable of creating a smoother tensile fracture surface, effectively enhancing the overall performance of the mortar. In Figure 9b, it can be observed that the PEF is pulled out rather than broken, indicating that it is still able to function as a bridge and maintain its bridging effect even after the matrix cracked [58]. Meanwhile, obvious scratches and hydrated products can be observed on the surface of the extracted PEF, and corresponding pull-out marks are also left in the matrix, indicating that the fibers have a strong adhesion to the matrix. This helps to increase the frictional resistance during the fiber extraction process and enhance the tensile properties of the mortar [59].

**Figure 10:**
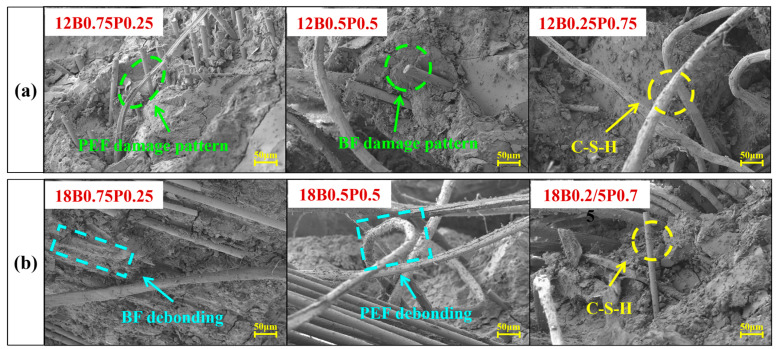
SEM images of mortars incorporating different blended fibers: (**a**) Mortars incorporating blended fibers with a length of 12 mm; (**b**) Mortars incorporating blended fibers with a length of 18 mm.

The microscopic morphologies of the mortars mixed with different types and lengths of BFs and PEFs are shown in Figure 10. It can be observed that the BF and PEF in the mortar are interlocked and intertwined with each other and form a three-dimensional spatial network structure within the mortar. This structure can effectively transfer and distribute stress, fully leveraging the synergistic reinforcing effect of the two types of fibers and significantly enhancing the strength and toughness of the mortar [60]. It is worth noting that in all samples, the extraction length of the PEF is greater than that of the BF. This is due to the different physical properties of the two types of fibers. As an inorganic fiber, BF has a relatively high elastic modulus, but its elongation at break is relatively low, making it more prone to brittle fracture during the pulling process, resulting in a relatively short pulling length. As a flexible fiber, PEF possesses excellent toughness and a high elongation at break. When subjected to force, it can undergo significant deformation without immediately breaking, thus enabling it to form a longer pull-out path within the mortar [50].

## 4. Mechanism Analysis

**Figure 11:**
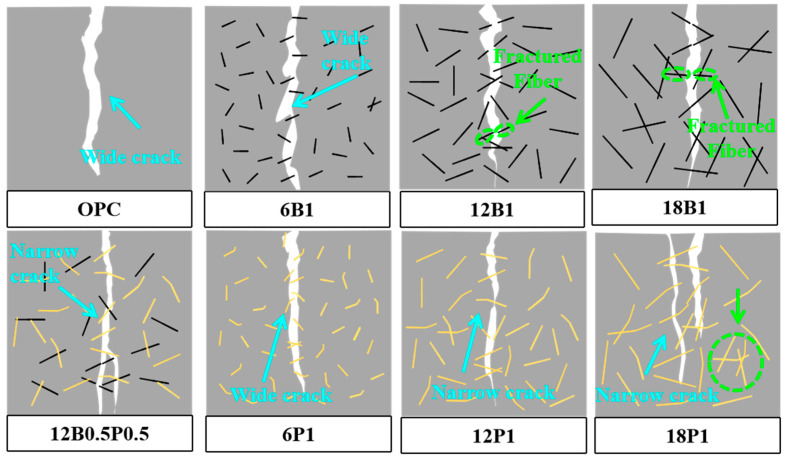
Mechanism diagram of the influence of single and blended incorporation of various lengths of BFs and PEFs on the microstructure of mortars.

The influence mechanisms of different lengths of BFs and PEFs, as well as their single and mixed additions, on the microstructure of mortars are shown in Figure 11. Under single BF addition, the dispersion of 6 mm BF is relatively good, but its ability to bridge microcracks is weak, making it difficult to effectively inhibit the expansion of cracks in the mortar. The 12 mm BF can not only be evenly dispersed in the mortar to form intermittent rigid supporting points, but also its surface can adhere to the C-S-H, enhancing the adhesion with the matrix and reducing the interface pores between fibers and matrix, which can effectively improve the tensile performance of the mortar and inhibit the initiation and expansion of mortar cracks [61,62]. The 18 mm BF is prone to agglomeration in the mortar, creating local weak areas, which leads to an increase in the number of interface pores and microcracks between the fibers and the matrix. This is because overly long BF is prone to fiber aggregation and overlapping, resulting in uneven fiber dispersion and an inability to be fully encapsulated by the cement slurry, thereby causing local defects in the matrix. The negative effect of the excessively long BF outweighs its reinforcing effect on the matrix, resulting in a decrease in the mechanical strength of the mortar [63]. Furthermore, in the case of single-doped PEF, it is difficult for the 6 mm PEF to form a three-dimensional flexible network in the mortar, and it has a poor ability to coordinate with the deformation of the matrix. The 12 mm PEF can be uniformly dispersed in the mortar and form a complete three-dimensional flexible reinforcing network, which helps to enhance the adhesion between fibers and the matrix [64]. Meanwhile, PEF can dissipate energy through stretching deformation or interface pull-out, effectively inhibiting the formation and development of microcracks in the mortar [65]. The 18 mm PEF tends to get entangled in the mortar, which disrupts the uniformity of the matrix and reduces its mechanical strengthening effect on the mortar as well as its ability to inhibit crack propagation.

In the blended fiber mortars, BF and PEF interweave with each other, forming a three-dimensional anisotropic network structure with complementary rigidity and flexibility. At this point, the high elastic modulus BF bears the main load and restricts the expansion of the crack width. The highly resilient PEF can dissipate energy through ductile deformation and interface slip, thereby inhibiting the initiation and propagation of cracks [46]. At the initial stage of loading, PEF primarily functions as a bridge, gradually transferring the stress to the matrix and the BF. As the cracks expand, the BF with higher elastic modulus will gradually bear higher stress, thereby delaying the further extension of the cracks. Meanwhile, PEF absorbs the fracture energy through the stretching deformation and extraction process, alleviating the stress concentration phenomenon. In the mixed fiber mortar with both BFs and PEFs of 12 mm, the two types of fibers can achieve a synergistic effect in terms of spatial configuration and mechanical division. At this point, the BF forms a rigid framework within the mortar, stabilizing the development path of cracks, while the PEF enhances the overall ductility and toughness of the mortar. The combined effect of these two factors can significantly enhance the multi-scale crack control and deformation capacity of the mortar. Furthermore, BF can optimize the structure of ITZ, and PEF can promote the deposition of hydration products on the fiber surface, thereby improving the density of the interface between fibers and the matrix. These two types of fibers can jointly enhance the mechanical properties of mortar and prevent the formation and development of mortar cracks.

## 5. Conclusions

This study investigated the effects of single-doping and blended-doping of different lengths of BFs and PEFs on the mechanical properties and microstructure of mortars. Based on experimental results, the following conclusions can be drawn:

The incorporation of fibers can effectively inhibit the drying shrinkage of mortars. The longer BF has a stronger resistance to drying shrinkage. The drying shrinkage of the mortar containing 18 mm BF is 19.2% lower than that of the mortar containing 6 mm BF. The shorter PEF has a stronger resistance to drying shrinkage. The drying shrinkage of the mortar containing 6 mm PEF is 34.4% lower than that of the mortar containing 18 mm PEF.

The mixed fibers exhibit a superior reinforcing effect in the mortar compared to the single fiber. When the fiber length is 12 mm and the ratio of BF/PEF is 1:1, the compressive strength of mortar at 28 d is 18.6% higher than that of the mortar without fiber, and the flexural strength of the mortar at 28 d is 56.1% higher than that of the mortar without fiber. When the fiber lengths are 12 mm and 18 mm, respectively, and the ratio of BF/PEF is 1:1, the splitting tensile strengths of the mortar at 28 d are 103.3% and 84.7% higher than that of the mortar without a fiber addition, respectively.

In the splitting tensile strain cloud diagram of the mortar, the crack width of the mortar with only BF added is wider. When longer PEFs (12 mm and 18 mm) are solely added to mortar, the crack width of the mortar significantly decreases. Meanwhile, when the PEF is 18 mm, two fine cracks appear in the mortar. The blending of BF and PEF has a significant inhibitory effect on the expansion of mortar cracks. As the proportion of PEF in the mixture gradually increases, the depth and width of the cracks in the mortar gradually decrease. The crack width of the mortar mixed with 18 mm fibers is smaller than that of the mortar mixed with 12 mm fibers.

The BF is randomly distributed in the crushed mortar in the form of single filaments, and no obvious wear marks are observed on the surface of the fibers. The end of the BF exhibits typical brittle fracture morphology. During the destruction process, PEF mainly exhibits fiber pull-out rather than fracture. Moreover, the surface of the pulled-out fibers can clearly show obvious scratches and adhered hydrated products. BF and PEF can form a three-dimensional spatial network structure within the matrix through mutual interlocking and intertwining, thereby enhancing the compactness of the mortar and increasing its mechanical strength.

This project has only investigated the influence of blending different lengths of BFs and PEFs, as well as the blending ratio, on the mechanical properties of the mortar. However, there is a lack of research on the influence of different dosages of these two types of fibers on the mechanical properties of the mortar. Furthermore, the durability of mortar reinforced with BFs and PEFs also requires further investigation. Therefore, in future research, we will conduct studies on the effects of different dosages of BF and PEF on the mechanical properties of mortar, as well as durability factors such as freeze-thaw resistance and chloride and sulfate ion resistance of mortar mixed with BF and PEF to provide guidance for the application of BF and PEF in actual engineering projects.

## Figures and Tables

**Table 1:** Chemical compositions of OPC (wt%).

Compositions	CaO	SiO_2_	Al_2_O_3_	Fe_2_O_3_	MgO	SO_3_	K_2_O	LOI
OPC	58.15	20.18	5.73	3.75	1.72	3.24	0.83	1.58

**Table 2:** Physical properties of fibers.

Fiber Types	Length (mm)	Diameter (μm)	Density (g/cm^3^)	Tensile Strength (MPa)	Elastic Modulus (GPa)	Elongation (%)
BF	6/12/18	15	2.65	2400	105	3.1
PEF	6/12/18	30	0.97	2900	60	15

**Table 3:** Mix proportions of fiber-reinforced mortars (kg/m^3^).

Samples	Cement (kg)	Sand (kg)	Water (kg)	Water Reducer (kg)	BF (%)	PEF (%)	Length (mm)
OPC	504	1512	252	5.04	0	0	0
6B1	504	1512	252	5.04	1.0	0	6
12B1	504	1512	252	5.04	1.0	0	12
18B1	504	1512	252	5.04	1.0	0	18
6P1	504	1512	252	5.04	0	1.0	6
12P1	504	1512	252	5.04	0	1.0	12
18P1	504	1512	252	5.04	0	1.0	18
12B0.75P0.25	504	1512	252	5.04	0.75	0.25	12
12B0.5P0.5	504	1512	252	5.04	0.5	0.5	12
12B0.25PE0.75	504	1512	252	5.04	0.25	0.75	12
18B0.75P0.25	504	1512	252	5.04	0.75	0.25	18
18B0.5P0.5	504	1512	252	5.04	0.5	0.5	18
18B0.25P0.75	504	1512	252	5.04	0.25	0.75	18

Note: 12B0.75P0.25 indicates the use of BF and PEF with a length of 12 mm each, and their volume fractions are 0.75% by volume and 0.25% by volume, respectively.

## Data Availability

The original contributions presented in this study are included in the article. Further inquiries can be directed to the corresponding authors.
